# Return to flight duty (RTFD) after posterior lumbar spine surgery for symptomatic lumbar disc herniation (LDH) and lumbar isthmic spondylolisthesis (LIS) in Chinese military pilots

**DOI:** 10.1186/s12891-024-07175-w

**Published:** 2024-01-20

**Authors:** Fengyuan Yang, Bowen Xie, Hongxing Zhang, Tianqi Li, Jian Mao, Zhiqiang Chen, Ye Peng, Tengfei Li, Siguo Sun, Jingyang Chen, Yufei Chen, Junjie Du

**Affiliations:** 1 Department of Orthopedics, Air Force Medical Center of PLA, Beijing, 100142 China; 2 Graduate School of Medicine, China Medical University, Shenyang, 110122 China; 3 The Fifth School of Clinical Medicine, Air Force Clinical College, Anhui Medical University, Anhui, 230032 China

**Keywords:** Military, Pilots, Lumbar surgery, Diskectomy, Fusion, Lumbar disc herniation, Lumbar isthmic spondylolisthesis

## Abstract

**Background:**

Symptomatic lumbar disc herniation (LDH) and lumbar isthmic spondylolisthesis (LIS) present significant challenges for military pilots, which may result in grounding if not effectively managed. Surgical treatment for LDH and LIS may offer a pathway to return to flight duty (RTFD), but recent data on this crucial topic is lacking. This study seeks to address this gap by investigating the RTFD outcomes among Chinese military pilots who have undergone lumbar spine surgery for symptomatic LDH and LIS.

**Methods:**

A retrospective review was conducted on active-duty military pilots who underwent isolated decompressive or fusion procedures at an authorized military medical center from March 1, 2007, to March 1, 2023. The analysis utilized descriptive statistics to examine demographic, occupational, surgical, and outcome data, with a particular focus on preoperative flight status, recommended clearance by spine surgeons, and actual RTFD outcomes and time.

**Results:**

Among the identified cases of active-duty military pilots with LDH or LIS treated by lumbar surgery (*n* = 24), 70.8% (17 of 24) consistently maintained RTFD status without encountering surgical complications or medical issues during the follow-up period. Of the seven pilots who did not RTFD, one retired within a year of surgery, two had anterior cruciate ligament injuries, three had residual radicular symptoms, and one had chronic low back pain. Excluding pilots who retired and did not RTFD for reasons unrelated to their lumbar conditions, the RTFD rate stood at 81.0% (17 of 21). The median time for recommended clearance by spine surgeons was 143.0 days (inter-quartile range, 116.5–196.0), while the median duration for actual RTFD attainment was 221.0 days (inter-quartile range, 182.0–300.0). The median follow-up post-lumbar surgery was 1.7 years (inter-quartile range, 0.4–2.9).

**Conclusion:**

Most military pilots diagnosed with symptomatic LDH and LIS can continue their careers and regain active-duty flight status following lumbar spine surgery, as reflected by the high RTFD rate. Lumbar spine surgery can successfully alleviate the physical constraints associated with spinal conditions, facilitating the return of military pilots to their demanding profession.

## Background

Lumbar disc herniation (LDH) is a condition where the herniated nucleus pulposus compresses the nerve roots after rupture of the annulus fibrosus, resulting in low back and leg pain as the primary symptom [1]. It is a prevalent issue among Chinese active-duty military aircrews, with an incidence rate of 16.1% [2]. Spondylolysis is a defect involving the pars interarticularis of the vertebrae, with a prevalence rate of about 10.9% among Chinese military flight crews [3]. Lumbar isthmic spondylolisthesis (LIS), in which one lumbar vertebra slips relative to the adjacent lower vertebra, can be considered a progression of spondylolysis. It can cause spinal stenosis and nerve compression [4]. While LDH and LIS symptoms may not significantly limit a typical career, they pose significant risks to military transport, helicopter, and high-performance jet pilots during extreme training and operational factors, including high G-forces, altered gravitational vectors, vibratory loads, and equipment interactions [5–7]. Acute or chronic low back pain, radiating pain, paresthesias, numbness and weakness in the legs, and reduced lumbar mobility caused by LDH or LIS can have significant adverse effects on pilots, manifesting as impaired attention and concentration, poor motor control, postural instability, inability to perform in-flight maneuvers, task interruption, and temporary or permanent grounding [5].

A majority of patients can achieve remission through nonoperative management, employing methods such as rest, physical therapy, nonsteroidal anti-inflammatory drugs, and traditional Chinese medicine. Lumbar surgery, encompassing isolated decompression and decompression with fusion, becomes an option for those with refractory symptoms that significantly limit function and correlate with radiographic pathology [8]. While surgery efficiently alleviates symptoms and manages complex conditions swiftly [1, 4], the inherent physical hazards tied to the procedure, including potential ineffectiveness, operative complications, and biomechanical changes to the lumbar spine, may impede patients from fully resuming active flying duties [6]. The impact of both the benefits and risks of surgical procedures on military pilots’ careers remains undetermined, particularly regarding whether lumbar spine surgery facilitates a return to flight duty (RTFD).

In recent decades, lumbar spine disorders have posed significant challenges to military interests, leading to dropout rates during training or early career termination [5]. A 2021 study revealed that 28 of the 366 (7.7%) Chinese military pilots permanently removed from active flight status in the past decade were affected by cervical and lumbar spine disorders [9]. In the realm of musculoskeletal disorders, 48 out of 134 (35.8%) Air Force and Army flight crew members grounded from 2003 to 2020 were attributed to lumbar spine diseases, including LDH, LIS, and discogenic low back pain [10]. The significance of these attrition rates is underscored by the substantial cost of training military pilots, estimated at around $9 million for an operational pilot [6] and approximately $15.2 million for a single fighter pilot [11]. Therefore, it is imperative for the military to understand the impact surgical intervention has on a pilot’s ability to RTFD.

In military medicine, evaluating service members’ return to active duty is a critical metric for assessing treatment effectiveness, as it typically signifies their complete functional recovery post-orthopedic procedures [12, 13]. Recently, return-to-duty rates for lumbar conditions post-simple decompression or decompression with fusion have been documented in the military population. However, these rates lack categorization by military occupation or the number of pilots [8, 14–21]. It is important to note that clearance for active duty in military pilots does not automatically extend to flight duty clearance. Aviators have unique criteria that demand more intensive therapeutic and rehabilitative outcomes than other military professions. In a 2007 study, Desviat et al. [22] examined ten Spanish fighter pilots who underwent lumbar spine surgery and achieved RTFD through single decompression or decompression with fusion. Their investigation excluded pilots of other aircraft, and they did not provide a comprehensive assessment of the proportion of military pilots who can resume RTFD after elective lumbar spine surgery and the associated timeframe. The need for a comprehensive understanding of how elective lumbar spine surgery affects the ability of military pilots to RTFD is highlighted by this gap. Although surgically treated LDH and LIS may allow for RTFD with waivers, it is important to note that these waivers are based on expert opinion rather than recent data. Therefore, this study aims to determine the RTFD of active military pilots who experience symptomatic LDH and LIS after elective lumbar spine surgery.

## Methods

We conducted a retrospective study at a single center to assess the RTFD after surgery for LDH or LIS within an active-duty military pilot population. Ethical approval was obtained from our hospital’s ethics committee, and a thorough search of our medical record retrieval system database was conducted for cases involving “lumbar disc herniation” and “lumbar isthmic spondylolisthesis.” Our methodology included carefully reviewing all operative reports to determine the diagnosis and the specific lumbar spine procedure performed.

Demographic data, encompassing age, branch of service, military occupation, and service status, were meticulously extracted from our Electronic Health Record (EHR) system. Identifying the active-duty pilot population relied on the occupational category data provided by the EHR system. To maintain the focus on active-duty pilots, individuals who had retired or separated from the military before their first follow-up visit were excluded from our analysis.

For each patient, a comprehensive retrospective chart review was conducted, capturing demographic information, preoperative flight status, type of aircraft flown (rotary or fixed wing), diagnosis, surgical approach (isolated neural decompression vs. decompression with fusion), specific surgical techniques, type of implants used, surgical levels, duration, and blood loss during surgery, length of hospital stay, postoperative complications, initiation of medical waiver and documentation of RTFD, time for waiver authority clearance for recommended RTFD, time for actual RTFD, and the follow-up period.

Continuous variables were presented as median and interquartile range (IQR), while categorical variables were reported as counts and percentages. Statistical analyses were performed using SPSS software (version 26.0, Chicago, USA).

## Results

### Preoperative basic information

Between March 1, 2007, and March 1, 2023, 308 cases of lumbar disc herniation (LDH) or lumbar isthmic spondylolisthesis (LIS) were surgically treated at the Air Force Medical Center, involving active duty, reserve, dependents, or veterans. Seven different spine surgeons from the Orthopedics Department conducted these procedures. Among the cases, 269 underwent isolated decompressive procedures, while 39 underwent fusion procedures. After screening for military occupation and active-duty status, 24 active-duty pilots underwent either isolated neural decompression or decompression with fusion, as detailed in Table 1.

**Table 1:** Summary of preoperative characteristics

Characteristic	Value
Number of pilots	24
Mean age at operation in years (IQR)	34.5 ( 30.0, 41.8)
Sex, no. (%)	
Male	24 (100.0)
Female	0 (0.0)
Hight, cm (range)	172.4 (165–178)
Weight, kg (range)	73.8 (54–90)
BMI, kg/m^2^ (range)	24.8 (19.8–30.1)
Flying time, hours (IQR)	2075.0 (1007.5, 3075.0)
Flight rating, no. (%)	
Third-grade	5 (20.8)
Second-grade	2 (8.3)
First-grade	16 (66.7)
Top-grade	1 (4.2)
Service, no. (%)	
Air Force	20 (83.3)
Army	4 (16.7)
Aircraft, no. (%)	
Fixed wing	19 (79.2)
Rotary wing	5 (20.8)
Tobacco use, no. (%)	
Yes	5 (20.8)
No	19 (79.2)
Main indication for surgery, no. (%)	
LDH	18 (75.0)
LIS	6 (25.0)

BMI, body mass index; LDH, lumbar disc herniation; LIS, lumbar isthmic spondylolisthesis. The median and inter-quartile range (IQR) present continuous variables. Categorical variables are expressed as counts and percentages

Within this specific pilot group, all 24 individuals were male, with a median age of 34.5 years (IQR, 30.0–41.8) and a median flying time of 2075.0 h (IQR, 1007.5–3075.0) at the time of surgery. The flight rating distribution among these pilots included five (20.8%) third-grade, two (8.3%) second-grade, sixteen (66.7%) first-grade, and one (4.2%) top-grade pilots. Regarding military branch distribution, 83.3% belonged to the Air Force, while 16.7% were from the Army. Fixed-wing and rotary-wing aircraft were operated by 79.2% and 20.8% of the pilots, respectively. In terms of smoking habits, 79.2% were non-smokers, and 20.8% were smokers. Before surgery, 18 (75.0%) of the 24 pilots were grounded for medical management and evaluation of LDH, while 6 (25.0%) were grounded for LIS.

### Clinical data

Among the 24 active-duty pilots, 16 (66.7%) underwent isolated neural decompression, while 8 (33.3%) opted for lumbar fusion in a total of 24 cases (Table 2). The procedures encompassed various single-level operations, including seven transforaminal lumbar interbody fusions (TILFs), six microendoscopic discectomies (MEDs), five percutaneous endoscopic lumbar discectomies (PELDs), four open fenestration discectomies (OFDs), and one unilateral biportal endoscopy (UBE). Notably, there was a singular instance of a contiguous two-level L3-5 TILF. The implant types utilized comprised eight pedicle screw systems (PSSs), cage implants, and 1 Wallis interspinous dynamic stabilization system (WIDSS) implant. Predominantly, level L4-5 (52.0%) was involved in most cases, followed by L5-S1 (44.0%) and L3-4 (4.0%).

**Table 2:** Operative summary

Parameter	No. (%)
Total cases	24
Procedure type	
Isolated neural decompression	16 (66.7)
Decompression with fusion	8 (33.3)
Surgical technique	
TILF	8 (33.3)
MED	6 (25.0)
PELD	5 (20.8)
OFD	4 (16.7)
UBE	1 (4.2)
Implant	
PSS and cage	8 (88.9)
WIDSS	1 (11.1)
Scopes of surgery	
1-level	23 (95.8)
2-level	1 (4.2)
Level	
L3-4	1 (4.0)
L4-5	13 (52.0)
L5-S1	11 (44.0)
Complication	
Chronic low back pain	1 (4.2)

TILF, transforaminal lumbar interbody fusion; MED, microendoscopic discectomy; PELD, percutaneous endoscopic lumbar discectomy; OFD, open fenestration discectomy; UBE, unilateral biportal endoscopic discectomy; PSS, pedicle screw system; WIDSS, Wallis interspinous dynamic stabilization system

The median length of lumbar simple decompression surgery lasted 120.0 min (IQR, 100.0–172.5) with a median blood loss of 45.0 ml (IQR, 21.3–80.0). In contrast, the median length for decompression with lumbar fusion surgery was 205.0 min (IQR, 186.3–235.0) with a median blood loss of 300.0 ml (IQR, 125.0–475.0). The post-surgery median hospital stay was 22.5 days (IQR, 14.3–31.8), and the median follow-up period after lumbar surgery was 1.7 years (IQR, 0.4–2.9).

Two pilots had a history of revision surgery. One pilot underwent a prior L4-5 PELD at a civilian hospital, initially receiving a flight waiver. However, a year later, due to adjacent-segment disc herniation necessitating an L3-5 TILF, he retired within a year after the second surgery. Another pilot had a previous L4-5 percutaneous lumbar discectomy (PLD) at a civilian hospital but was initially denied a flight waiver due to residual left ankle pain lasting eight months. Subsequently, experiencing a recurrent disc herniation at L4-5, this pilot underwent an L4-5 MED at our hospital and was granted a waiver for a fixed-wing aircraft with an ejection seat 157 days after the revision surgery. Moreover, two pilots who initially underwent single-level L5-S1 TILF for LIS at our medical center personally requested the removal of the PSS at 662 and 964 days after the initial surgery, respectively. Following this, they were granted waivers to operate ejection seat aircraft.

### Return to flight duty (RTFD)

Among the 17 postoperative pilots who consistently maintained RTFD status without encountering surgical complications or medical issues during the follow-up period, the distribution by surgery type was as follows: 5 underwent single-level TILFs, 4 underwent single-level MEDs, 4 underwent single-level PELDs, 3 underwent single-level OFDs, and 1 underwent single-level UBE (Table 3). One pilot retired within a year after surgery, while six others were not eligible for retirement and did not resume RTFD after surgery (Table 4). Among these six pilots, two were disqualified due to anterior cruciate ligament (ACL) injuries unrelated to lumbar conditions. The remaining four pilots were denied flight waivers due to symptoms associated with lumbar disorders, leading them to undergo evaluations by military aeromedical boards, resulting in permanent grounding. Within this group, three pilots with LDH continued experiencing residual radicular symptoms in their legs that did not improve with surgery (one reported numbness in the left foot, another had decreased sensation and strength in the left calf, and the third experienced pain in the left foot). The remaining pilot, preoperatively diagnosed with LIS, developed chronic low back pain after surgery and was subsequently diagnosed with failed back surgery syndrome (FBSS), necessitating alternative therapies.

**Table 3:** Characteristics of patients with successful return to flight duty (RTFD)

Age	Rating	Diag	Type of surgery	Op	Level	Implant	Time to SSR (days)	Time to RTFD (days)	Rotary or Fixed Wing	Aircraft
34	1st	LDH	Isolated decompression	MED	L4-5	—	114	159	Fixed	Jet trainer
35	1st	LDH	Isolated decompression	MED	L5-S1	—	95	189	Fixed	Jet trainer
28	3rd	LDH	Isolated decompression	MED^*^	L4-5	—	157	177	Fixed	Fighter
42	1st	LDH	Isolated decompression	MED	L4-5	—	119	156	Rotary	Helicopter
30	2nd	LDH	Isolated decompression	PELD	L4-5	—	165	201	Fixed	Fighter
34	1st	LDH	Isolated decompression	PELD	L4-5	—	143	221	Rotary	Helicopter
25	3rd	LDH	Isolated decompression	PELD	L5-S1	—	197	299	Fixed	Fighter
45	1st	LDH	Isolated decompression	PELD	L4-5	—	131	162	Fixed	Transport
41	1st	LDH	Isolated decompression	OFD	L4-5	—	98	187	Fixed	Fighter
30	1st	LDH	Isolated decompression	OFD	L4-5	WIDSS	170	259	Fixed	Transport
46	1st	LDH	Isolated decompression	OFD	L4-5	—	112	234	Rotary	Helicopter
40	1st	LDH	Isolated decompression	UBE	L4-5	—	130	195	Fixed	Jet trainer
34	1st	LIS	Decompression with fusion	TILF	L5-S1	PSS and cage	558	708	Fixed	Fighter-bomber
31	1st	LIS	Decompression with fusion	TILF	L5-S1	PSS (removal) and cage	774	849	Fixed	Fighter-bomber
27	3rd	LIS	Decompression with fusion	TILF	L5-S1	PSS (removal) and cage	1077	1233	Fixed	Fighter-bomber
42	Top	LIS	Decompression with fusion	TILF	L5-S1	PSS and cage	195	226	Fixed	Transport
44	1st	LIS	Decompression with fusion	TILF	L5-S1	PSS and cage	123	301	Fixed	Fighter

Diag, diagnosis; Op, operation; SSR, spine surgery recommendation; RTFD, return to flight duty; LDH, lumbar disc herniation; LIS, lumbar isthmic spondylolisthesis; TILF, transforaminal lumbar interbody fusion; PSS, pedicle screw system; MED, microendoscopic discectomy; PELD, percutaneous endoscopic lumbar discectomy; OFD, open fenestration discectomy; WIDSS, Wallis interspinous dynamic stabilization system; UBE, unilateral biportal endoscopy; ^*^Revision surgery

**Table 4:** Characteristics of retired pilot and patients who failed to return to flight duty (RTFD)

Age	Rating	Diag	Type of surgery	Op	Level	Implant	Time to SSR (days)	Reason for Failure	Rotary or Fixed Wing	Aircraft
49	1st	LDH	Decompression with fusion	TILF^*^	L3-5	PSS and cage	191	Retire	Fixed	Transport
38	1st	LDH	Decompression with fusion	TILF	L4-5	PSS and cage	210	ACL injury	Rotary	Helicopter
25	3rd	LDH	Isolated decompression	OFD	L5-S1	—	362	ACL injury	Fixed	Fighter
31	2nd	LDH	Isolated decompression	MED	L5-S1	—	—	Residual symptoms	Fixed	Transport
22	3rd	LDH	Isolated decompression	MED	L4-5	—	—	Residual symptoms	Fixed	Fighter
38	1st	LDH	Isolated decompression	PELD	L5-S1	—	—	Residual symptoms	Rotary	Helicopter
38	1st	LIS	Decompression with fusion	TILF	L5-S1	PSS and cage	—	FBSS	Fixed	Jet trainer

Diag, diagnosis; Op, operation; SSR, spine surgery recommendation; LDH, lumbar disc herniation; LIS, lumbar isthmic spondylolisthesis; TILF, transforaminal lumbar interbody fusion; PSS, pedicle screw system; ACL, anterior cruciate ligament; MED, microendoscopic discectomy; PELD, percutaneous endoscopic lumbar discectomy; OFD, open fenestration discectomy; ^*^Revision surgery

Among the 24 pilots studied, 20 (83.3%) received recommendations for RTFD from the spinal surgeon (Table 5). It’s important to note that no pilot obtained a flight waiver without prior clearance from the spine surgeon. Excluding the pilot who retired within a year of surgery, 17 of the remaining 23 pilots (73.9%) resumed RTFD. If we exclude the two pilots who didn’t resume flying due to reasons unrelated to lumbar diseases, the overall RTFD rate increases to 81.0% (17 of 21). When considering different procedure types, the RTFD rate was 80.0% for isolated decompression and 83.3% for decompression with fusion. In terms of surgical indication, the RTFD rate was 75.0% for the LDH group and 100.0% for the LIS group. Stratifying by the follow-up period, the RTFD rate within 12 months post elective lumbar surgery was 66.7% (75.0% in the LDH group and 40.0% in the LIS group, 80.0% in the isolated decompression group and 33.3% in the decompression with fusion group).

**Table 5:** Summary of rates of return to flight duty (RTFD)

Parameter	Value
Recommended for unrestricted active flight duty by spinal surgeon	20 of 24 (83.3%)
Recommended for unrestricted active flight duty by spinal surgeon (LDH)	14 of 18 (77.8%)
Recommended for unrestricted active flight duty by spinal surgeon (LIS)	5 of 6 (83.3%)
Retired within one year of operation	1 of 24 (4.2%)
Did not return to flight status	6 of 24 (25.0%)
LD-induced DQ	4 of 6 (66.7%)
Non-LD-related DQ	2 of 6 (33.3%)
RTFD	17 of 24 (70.8%)
RTFD without retired pilot	17 of 23 (73.9%)
RTFD without retired pilot & non-LD DQ	17 of 21 (81.0%)
RTFD without retired pilot & non-LD DQ (LDH)	12 of 16 (75.0%)
RTFD without retired pilot & non-LD DQ (LIS)	5 of 5 (100.0%)
RTFD without retired pilot & non-LD DQ (Isolated decompression)	12 of 15 (80.0%)
RTFD without retired pilot & non-LD DQ (Decompression with fusion)	5 of 6 (83.3%)
12-month RTFD without retired pilot & non-LD DQ	14 of 21 (66.7%)
12-month RTFD without retired pilot & non-LD DQ (LDH)	12 of 16 (75.0%)
12-month RTFD without retired pilot & non-LD DQ (LIS)	2 of 5 (40.0%)
12-month RTFD without retired pilot & non-LD DQ (Isolated decompression)	12 of 15 (80.0%)
12-month RTFD without retired pilot & non-LD DQ (Decompression with fusion)	2 of 6 (33.3%)

LD, lumbar diseases; DQ, disqualification. Categorical variables are expressed as counts and percentages

Regarding the timeline for spine surgeon recommendations and pilot resumption of RTFD, for the 20 pilots who received clearance from the spine surgeon, irrespective of whether they resumed actual flying or not, the recommendation for RTFD came after a median time of 161.0 days (IQR, 120.0–206.8) (Table 6). Among the 17 pilots who successfully resumed RTFD, the median time for spine surgeon approval was 143.0 days (IQR, 116.5–196.0), and the median time for actual RTFD was 221.0 days (IQR, 182.0–300.0). When categorized by surgery type, for isolated decompression (*n* = 12), the median time for spine surgeon approval was 130.5 days (IQR, 112.5–163.0), and the median time for actual RTFD was 192.0 days (IQR, 165.8–230.8). In the case of fusion surgery (*n* = 5), the median time for spine surgeon approval was 558.0 days (IQR, 159.0–925.5), and the median time for actual RTFD was 708.0 days (IQR, 263.5–1041.0). When excluding the two pilots who requested the removal of PSSs, the median time for spine surgeon approval was 195.0 days (IQR, 159.0–376.5), and the median time for actual RTFD was 301.0 days (IQR, 263.5–504.5).

**Table 6:** Summary of time to return to flight duty (RTFD)

Parameter	Value
Time to recommended RTFD	161.0 (120.0, 206.8)
Time to recommended RTFD without retired pilot & non-LD DQ	143.0 (116.5, 196.0)
Time to recommended RTFD without retired pilot & non-LD DQ (Isolated decompression)	130.5 (112.5, 163.0)
Time to recommended RTFD without retired pilot & non-LD DQ (Decompression with fusion)	558.0 (159.0, 925.5)
Time to recommended RTFD without retired pilot & non-LD DQ (Decompression with fusion/preservation of PSS)	195.0 (159.0, 376.5)
Time to RTFD without retired pilot & non-LD DQ	221.0 (182.0, 300.0)
Time to RTFD without retired pilot & non-LD DQ (Isolated decompression)	192.0 (165.8, 230.8)
Time to RTFD without retired pilot & non-LD DQ (Decompression with fusion)	708.0 (263.5, 1041.0)
Time to RTFD without retired pilot & non-LD DQ (Decompression with fusion/preservation of PSS)	301.0 (263.5, 504.5)

LD, lumbar diseases; DQ, disqualification; PSS, pedicle screw system. The median and inter-quartile range (IQR) present continuous variables

## Discussion

Our study findings highlight the significant positive impact of lumbar spine surgery on military pilots grappling with LDH and LIS. Among our sample of 24 active-duty pilots, the RTFD rate was 70.8%. Excluding pilots due to retirement and those who did not RTFD for reasons other than lumbar diseases, the overall RTFD rate among the military pilot population reached 81.0% post-lumbar surgery. This encompassed various surgical procedures, be it decompression or fusion. These RTFD rates could serve as crucial guidance for military surgeons and primary care physicians, especially flight surgeons, enabling them to advise military pilots affected by symptomatic LDH and LIS about the viability of surgical interventions and the possibility of resuming active flight duty.

The study found an overall RTFD rate of 81.0% after lumbar surgery, which is relatively high compared to other reported results for return to active duty not categorized by military occupation, which ranged from 52.4 to 100% [8, 14–21]. This high RTFD rate demonstrated is of great value to military aviation, showing that many pilots with LDH and LIS can successfully resume active flight duty without limitations following surgical treatment. Lumbar spine surgery effectively alleviates symptoms like back and leg pain, numbness, paresthesia, and leg weakness. This is crucial as it restores the physical capabilities necessary for pilots to handle extreme conditions characterized by high G-forces, altered gravitational vectors, vibratory loads, and complex equipment interactions [5]. Given the substantial investment in training military pilots, preventing career termination due to medical conditions becomes imperative. Lumbar spine surgery emerges as a pivotal intervention, preserving experienced pilots and ensuring the operational readiness of military aviation units. Beyond achieving the desired outcomes of surgery, the high RTFD rate can be attributed to the military’s heightened commitment to this goal. This commitment manifests through increased access to medical resources, policy support, and encouragement provided to pilots to resume their duties, distinguishing it from other military occupations. This support not only aids in their recovery but also underscores the esteemed nature of their profession, contributing positively to their successful re-engagement in duty.

The results also underscore the importance of personalized medical care for military personnel. Each case of LDH and LIS is unique, and tailoring treatment to the individual needs of pilots appears to contribute to a favorable outcome. This individualized approach is likely reflected in the specific surgical technique used, such as isolated decompression procedures (MED, PELD, OFD, and UBE) and decompression with fusion (TILF). However, it is important to note that surgical techniques may impact the pilot’s postoperative outcome, such as the rate of RTFD at a given time. In our report on military pilots who underwent elective lumbar surgery, including isolated decompressive and fusion procedures, we found a 12-month RTFD rate of 66.7%. This is consistent with the findings of Lunsford et al. [20], who did not stratify by military occupation and reported a 12-month return to duty rate of 64%. However, differences arise when comparing isolated decompression and decompression with fusion procedures. Our study found that 80.0% of patients who underwent isolated decompression procedures were able to achieve RTFD within 12 months. In contrast, the situation for our fusion group is less optimistic, with only 33.3% of patients who underwent TILF reaching RTFD within 12 months. The difference in 12-month RTFD rates may be due to the fact that RTFD criteria for military pilots typically require complete radiographic fusion in the TILF population. Additionally, removing the pedicle screw system, whether due to personal requests or hardware failure, can delay RTFD for pilots undergoing fusion surgery.

Seemingly, isolated decompression may allow these pilots to reach RTFD sooner than fusion procedures. This is reflected not only by the difference in the 12-month RTFD rates mentioned above but also by the actual time it takes to complete RTFD. The study showed that the median time of actual RTFD in the isolated decompression group was 192.0 days (IQR, 165.8–230.8), which was shorter than the median time of 708.0 days (IQR, 263.5–1041.0) in the lumbar fusion surgery group. However, it is important to exercise caution when considering this view. Spinal fusion offers advantages over decompression alone in cases involving combined multi-segmental lesions, spinal deformity, and lumbar instability [23, 24], particularly in the case of lumbar fractures, which may result in an inability to return to duty on military aircraft following an accident [25]. Further studies are necessary to clarify the impact of specific surgical techniques on the RTFD outcome and time of military pilots. In particular, minimally invasive techniques have shown promising prospects in the field of military medicine [21].

Although the timing of individual military pilots’ return to flying may vary, our study has shown that lumbar spine surgery is a positive factor in enabling most pilots diagnosed with symptomatic LDH and LIS to continue their challenging roles. However, an important consideration arises from the cases in our study, which illustrate instances in which pilots have faced challenges in RTFD due to their inability to meet the demanding waiver criteria (Table 7) [26, 27]. Within our study group, three pilots with LDH experienced persistent radicular symptoms in their legs that did not improve with surgery, and another patient with LIS who underwent TILF developed chronic low back pain postoperatively and was diagnosed with failed back surgery syndrome (FBSS). These adverse events prevented these pilots from passing medical assessments and resuming their flying duties.

**Table 7:** Chinese Military Pilot Medical Waiver Guidelines for Lumbar Disc Herniation (LDH) and Lumbar Isthmic Spondylolisthesis (LIS) [26, 27]

Disease	LDH	LIS
Last update	April 2022	June 2021
Ground observation period	1 month after conservative treatment 3 months after open or minimally invasive isolated decompression Spinal fusion surgery requires evidence of bony fusion and confirmation of stability on dynamic (flexion-extension) radiographs	3 months after conservative treatment Spinal fusion surgery requires evidence of bony fusion and confirmation of stability on dynamic (flexion-extension) radiographs
Waiver consideration	Asymptomatic LDH or LDH symptoms resolved No residual motor/sensory/reflex deficits No lumbar motion restriction Pass physical fitness tests without precipitating the disease Pass rehabilitation assessments Clearance by a spine surgeon to return to flight	Asymptomatic spondylolysis or asymptomatic grade I spondylolisthesis with no or mild spinal stenosis or LIS symptoms resolved No residual motor/sensory/reflex deficits No lumbar motion restriction Pass physical fitness tests without precipitating the disease Pass rehabilitation assessments Clearance by a spine surgeon to return to flight
Additional remark	High-performance aircraft pilots must meet specific physical standards and acceleration endurance requirements without inducing symptoms and signs Fighter and attack helicopter pilots with only residual mild symptoms after treatment that do not interfere with daily life and groundwork may be granted flight waivers for dual-control aircraft	

Residual symptoms and FBSS can cause ongoing discomfort in the lower back, legs, or buttocks, significantly impacting a pilot’s ability to cope with the physical demands of flying. The vibrations and forces experienced during flight can worsen the pain, making it difficult to maintain focus and alertness. Furthermore, limited mobility and flexibility in the lumbar spine can hinder a pilot’s ability to maneuver within the cockpit and access controls during flight operations, compromising their effectiveness and safety. Additionally, persistent symptoms and FBSS can lead to neurological impairments such as numbness, tingling, or weakness, posing a significant threat to flight safety. These impairments can affect a pilot’s control over the aircraft’s pedals, their ability to make precise movements, and their responsiveness to flight conditions. Furthermore, the functional limitations resulting from residual symptoms and FBSS can impede a pilot’s performance of physically demanding tasks and emergency procedures. Swift reactions to unforeseen situations are crucial for pilot competence, and physical impairments put the aircraft and crew at risk. Chronic pain and the limitations associated with residual symptoms and FBSS can also have a profound psychological impact on pilots, leading to negative emotions, decreased motivation, and distress. This psychological distress further impedes their ability to handle job demands and exacerbates difficulties, posing a risk to their safety [26, 27].

In addition, even after successful lumbar surgery and optimal symptom relief, pilots may continue to face challenges in RTFD due to other medical conditions, such as anterior cruciate ligament (ACL) injuries and ACL reconstruction (ACLR). The precision, coordination, and stability required for flying can be compromised by knee joint limitations resulting from ACL injury or recovery from ACLR. If these limitations affect a pilot’s ability to control the aircraft or perform required maneuvers safely, the pilot may be deemed unfit for active flight duty [12]. Thus, future research priorities in these contexts will focus on identifying and reducing the challenges that affect pilots’ ability to safely fly aircraft after lumbar surgery, as well as positively coping with threats posed by other medical problems.

This study provides essential information on spinal disorders for military healthcare providers, aeromedical researchers, and force commanders. However, it is important to consider the study’s limitations. Firstly, the study has a retrospective design and a relatively small sample size, which warrants caution when generalizing the findings to a larger population. To establish more robust conclusions, further research with larger sample sizes and prospective designs would be beneficial to confirm and broaden these results. Secondly, it is worth noting that the study needs more information on the follow-up process with the flight surgeon, who maintains regular contact with the pilot after the spine surgeon recommends RTFD. Additionally, there needs to be long-term follow-up data regarding the impact of lumbar surgery on pilots’ careers. Therefore, improving follow-up models for military pilot patients and obtaining extended-term follow-up information are necessary for a more comprehensive understanding. Thirdly, incorporating more specific outcome scores would enhance the study. Comparing RTFD rates with validated outcome measures specific to each surgery type would provide a clearer picture of RTFD as a functional outcome measure, and this area should be a focus of future research. Finally, due to the multiple treatment options and variations among treating surgeons, it is challenging to draw definitive conclusions regarding the optimal surgical plan for the military pilot population. Future prospective randomized controlled trials are needed to address these issues more comprehensively.

## Conclusions

The high RTFD rate of pilots after lumbar surgery highlights the positive impact of this intervention on their pivotal roles within the military aviation domain. The specific surgical techniques employed for treating LDH and LIS may impact the overall outcome and the time required for pilots to regain their flight status. Efforts should be made to prevent and address potential adverse factors that may impede a pilot’s ability to safely operate military aircraft after surgery, which is an important area for future research.

## Data Availability

The data sets used and analyzed during the current study are available from these corresponding authors upon reasonable request.

## Abbreviations

RTFD: Return to flight duty
BMI: Body mass index
IQR: Inter-quartile range
LDH: Lumbar disc herniation
LIS: Lumbar isthmic spondylolisthesis
TILF: Transforaminal lumbar interbody fusion
MED: Microendoscopic discectomy
PELD: Percutaneous endoscopic lumbar discectomy
OFD: Open fenestration discectomy
UBE: Unilateral biportal endoscopic discectomy
PSS: Pedicle screw system
WIDSS: Wallis interspinous dynamic stabilization system
LD: Lumbar diseases
DQ: Disqualification
Diag: Diagnosis
Op: Operation
SSR: Spine surgery recommendation
ACL: Anterior cruciate ligament
ACLR: Anterior cruciate ligament reconstruction
